# Lateral Cortical Fixation as the Optimal Strategy for Achieving Stability in Rib Fractures: A Patient-Specific Finite Element Analysis

**DOI:** 10.3390/bioengineering12060594

**Published:** 2025-05-31

**Authors:** Xiang Zhang, Xuejun Lan, Wang Shen, Qinghua Zhou

**Affiliations:** 1 Department of Thoracic Surgery, West China Hospital, Sichuan University, Chengdu 610041, China; 2 Lung Cancer Center, West China Hospital, Sichuan University, Chengdu 610041, China; 3 Health Management Center, General Practice Medical Center, West China Hospital, Sichuan University, Chengdu 610041, China

**Keywords:** rib fracture, surgical stabilization of rib, biomechanics, finite element analysis

## Abstract

The surgical stabilization of rib fractures helps maintain chest wall stability and reduces respiratory complications. This study aimed to identify the key biomechanical parameters for evaluating the stability of rib fracture fixation using finite element analysis (FEA) and compare four rib fixation configurations—intramedullary rib splint (IRS), locking plate (LP), claw-shape plate, and intrathoracic plate (IP)—using biomechanical analysis. Forty patient-specific FEA models of fourth-rib fractures were constructed using the computed tomography scans of 10 patients. Maximum implant displacement (MID), maximum rib fracture displacement, maximum implant von Mises stress (MIVMS), maximum rib von Mises stress, maximum rib strain, and maximum interfragmentary gap (MIG) were assessed by simulating the anterior and posterior loads on the ribs during postoperative frontal collision. The fixation stabilities were evaluated using entropy scores. MIVMS, MIG, and MID exhibited the highest weighting coefficients. Lateral cortical fixation strategies, particularly LP configuration, demonstrated superior biomechanical performance compared with IRS and IP systems. The composite score of the LP was significantly higher than that of the other modalities. MIVMS, MIG, and MID were identified as critical parameters for evaluating the rib fracture fixation stability, and the lateral cortical fixation strategy (LP) enhanced the structural stability of rib fracture fixation.

## 1. Introduction

Rib fractures occur in up to 43% of cases involving blunt chest injuries [1], with incidence rates increasing by 64.1% since 1990 [2]. Flail chest, resulting from multiple rib fractures, often leads to complications such as abnormal chest wall movement, pain, and decreased respiratory function. Subsequently, this increases the risk of pulmonary infection and acute respiratory distress syndrome, contributing to a mortality rate of up to 33% [3]. For most patients with rib fractures, conservative treatment options including multimodal pain management, inhaled bronchodilators, and ventilator support, are the treatments of choice [4]. However, conservative treatment is associated with an increased risk of complications, including chronic pain, chest wall deformity, and bone nonunion [5,6]. The surgical stabilization of rib fractures (SSRF) significantly improves the short- and long-term prognoses of patients compared with conservative treatment. Specifically, the SSRF plays a key role in promoting fracture healing, relieving pain, restoring chest wall stability, and reducing mortality [7,8].

With advances in implant design and surgical techniques, the fixation techniques for rib fractures are becoming increasingly diverse. Intramedullary splints, claw plates, and locking plates are widely used to treat rib fractures [9]. Recently, minimally invasive rib fracture fixation techniques, assisted by thoracoscopy, have been introduced. These techniques offer the advantages of minimizing soft tissue injury and accelerating the patient’s recovery [10]. Regardless of the fixation system used, the rib fracture stabilization methods have historically been divided into two categories: cortical and intramedullary fixation [11]. Although biomechanical experiments and clinical studies have reported the mechanical properties and clinical outcomes of different rib fracture fixation devices, a consensus on the optimal fixation protocol remains lacking [12].

Fracture fixation stability is a crucial biomechanical factor that affects fracture healing and clinical outcomes [13]. Improvement in the respiratory function of the patient is strongly correlated with fracture fixation stability. Given the challenges of directly studying the biomechanical metrics of fracture stability and implant failure risk directly in vitro or in vivo, finite element analysis (FEA) has emerged as a reliable method for evaluating these parameters [14]. In analyzing the complex mechanical behavior related to the primary stability of fracture fixation structures, FEA offers distinct advantages over other computational and experimental methods [15]. The present study aimed to numerically investigate the surgical fixation of rib fractures in specific patients using FEA, assess the biomechanical stability of four different fixation modalities, and provide a biomechanical reference for selecting the appropriate implantation protocols for the SSRF.

## 2. Materials and Methods

### 2.1. Participants

The study protocol was reviewed and approved by the Biomedical Research Ethics Committee of our hospital (IRB #2023-776). In this study, we retrospectively analyzed the preoperative chest CT scans of patients who suffered rib fractures and underwent surgical treatment. A chest CT is a routine preoperative examination required for patients. Given the retrospective nature of the study, the ethics committee waived the requirement for written informed consent.

The minimum sample size required to compare the four modalities was determined to be 10 (β = 0.2, α = 0.05) based on the power analysis. Chest computed tomography (CT) images of 10 patients were analyzed (Table A1). None of the patients had a history of bone pathology (including metastases, spinal fractures, or metabolic or hematologic disorders) at the time of the CT scan. All CT examinations were performed using three multidetector-row CT scanners (SOMATOM SENSATION 64, Siemens Healthcare, Mumbai, India) with a spatial resolution of 13.7 Lp/cm @ 10% MTF.

### 2.2. FEA Modeling

Chest CT images with a layer thickness of 0.8 mm were imported into Mimics 21.0 (Materialise Group, Leuven, Belgium) to reconstruct patient-specific rib models. The models were then imported into Geomagic Wrap 2021 (Geomagic, Rock Hill, SC, USA) for patch construction and surface fitting and subsequently exported in an stp. format. The FE modeling of a single rib exhibits the unique advantage of representing local cortical bone changes, providing insights into rib mechanical properties and damage prediction [16]. The fourth rib is widely used in biomechanical studies and comparable data are available [17]. Consequently, the fourth rib was selected to construct the solid rib model in this study. Actual collisions and rib anterior-posterior loading experiments have shown that fractures are mainly concentrated in the front-lateral region of the chest wall and mid-ribs [18,19]. Therefore, we performed an osteotomy from the midpoint of the rib cartilage junction to the transverse process of the posterior thoracic vertebrae according to the osteotomy scheme proposed by Prins et al. [12]. Ten patient-specific models of transverse simple rib fractures with the same fracture morphology and location were constructed using SolidWorks 2021 software (DS Solidworks Corp., Waltham, MA, USA).

**Figure 1:**
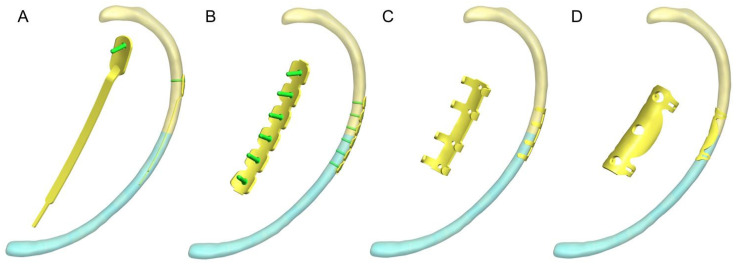
Schematic diagram of four implants for rib fractures: (**A**) The intramedullary splint fixation group; (**B**) the locking plate group; (**C**) the claw-shape plate group; (**D**) the intrathoracic plate fixation group.

Four internal fixation models—the Synthes intramedullary rib splint (IRS; DePuy Synthes, West Chester, PA, USA), the MatrixRIB locking plate (DePuy Synthes, West Chester, PA, USA), the memory alloy embracing plate (Changzhou Huasen Medical Device, Changzhou, China), and the transthoracic memory alloy rib coaptation board (Lanzhou Seemine Shape Memory Alloy Co., Ltd., Lanzhou, China)—were constructed using SolidWorks based on the manufacturer-supplied dimensions. Subsequently, the rib fracture models were stabilized using four different implants according to the fixation protocols used in previous studies. In the IRS fixation group, the cortical bone was drilled 30 mm from the fracture end, and a Synthes intramedullary rib splint was inserted across the fracture end [20]. The proximal end of the intramedullary splint was fixed using a locking screw, and the distal end remain unfixed. Fracture stabilization was achieved using intramedullary splint stiffness (Figure 1A). In the locking plate (LP) fixation group, rib fractures were repaired using the Synthes MatrixRIB system. The LP was placed on the lateral cortex of the rib, and the fracture was stabilized with three locking screws at each end (Figure 1B). In the claw-shape plate (CSP) fixation group, a memory alloy embracing plate was used to fit the lateral edge of the ribs. The rib was fixed using the hugger clips (Figure 1C) [21]. In the intrathoracic plate (IP) fixation group, a memory alloy rib splint was used to tightly fit the medial edge of the rib to achieve stable fixation using a clamping mechanism on the board to simulate video-assisted thoracoscopic surgical fixation of rib fractures (Figure 1D) [22].

### 2.3. FE Parameter Setting

The established models of rib fractures using four different strategies were imported into ANSYS Workbench 2020R2 (ANSYS, Canonsburg, PA, USA) for FEA. All the models were assumed to be linear elastic materials. The bone model was determined to be anisotropic and each finite element cell in the model exhibited unique mechanical properties. The apparent density (ρ), modulus of elasticity (E), and Poisson’s ratio (ν) of the cortical and cancellous bone of the ribs were calculated using the Mimics and ANSYS software programs and assigned based on the following equations [23]

ρ (g/cm^3^) = 0.007764 HU − 0.056148

E (GPa) = 10.5ρ^2.29^, and ν = 0.3

where HU is the Hounsfield Unit. The IRS and LP were modeled using a titanium alloy (Ti–6AL–7Nb) having E and ν of 110 GPa and 0.35, respectively [24], whereas the CSP and IP were modeled using a Ti–Ni memory alloy having E and ν of 65.84 GPa and 0.33, respectively [25]. The mesh size of the LP fixation structure was set to 0.4, 0.6, 0.8, and 1 mm for the mesh convergence analysis. A mesh size of 0.8 mm produced a mesh-independent solution, with variations in mesh size resulting in a reduced computation time and less than a 5% change in the maximum von Mises stress (Table A2). Therefore, the model was meshed using hexahedral elements with a mesh size of 0.8 mm [26]. The numbers of mesh elements and nodes for all the models are listed in Table 1. Frictional contact was defined between the fracture surfaces of the anterior and posterior ends of the rib and between the bone and the implant, with friction coefficients of 0.46 and 0.30, respectively. In contrast, the screw-plate and screw-bone contacts were defined as bonded contacts [27].

**Figure 2:**
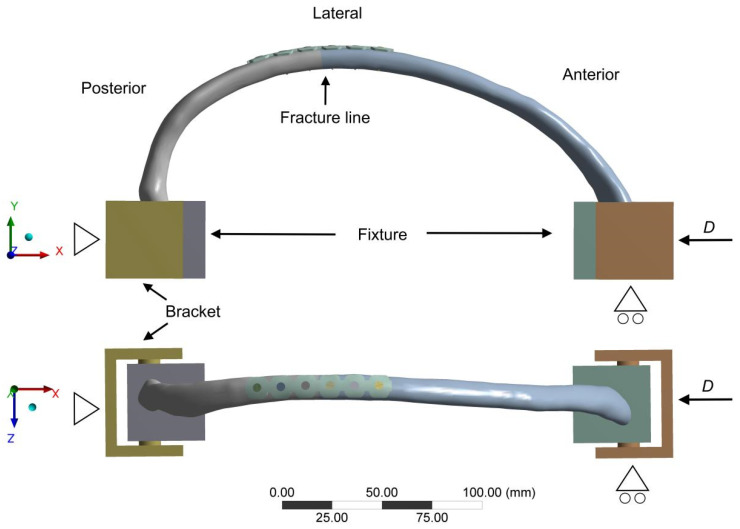
The front and top views of the rib fracture fixation model boundary condition setup. “*D*” stands for displacement.

**Figure 3:**
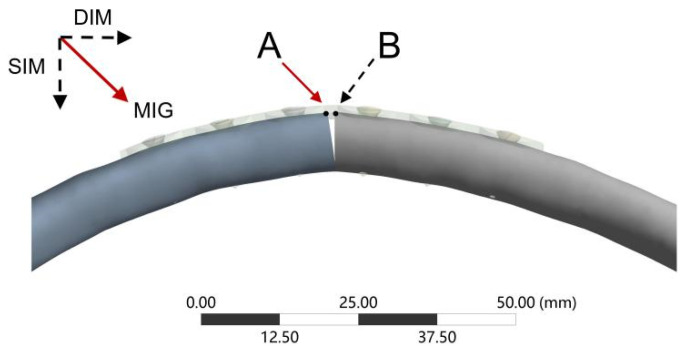
A schematic diagram of the maximal interfragmentary gap measurement. The maximal interfragmentary gap (MIG) was defined as the vector sum of SIM (Shear interfragmentary motion) and DIM (Detached interfragmentary motion). A and B were the distal and proximal fracture surface paired nodes, respectively. The SIM and DIM were obtained by calculating the absolute value of the difference between the final positions of points A and B in the shear and detached directions.

We simulated the anteroposterior loads experienced by the ribs during frontal collisions in the chest of postoperative patients with rib fractures to investigate the loads sustained under extreme conditions. As shown in Figure 2, the posterior end of the fourth rib near the transverse process was fixed, and a displacement of 14 mm was applied to the anterior end of the rib (sternal end), allowing a rotation around the vertical axis of the loading plane according to the loading scheme in the biomechanical experiments of Li et al. [17]. Based on previous studies, biomechanical parameters such as maximum implant displacement (MID), maximum rib fracture displacement (MRFD), maximum implant von Mises stress (MIVMS), maximum rib von Mises stress (MRVMS), maximum rib strain (MRS), and maximum interfragmentary gap (MIG) (Figure 3) were recorded. Each of these six parameters may be critical in assessing the stability of rib fracture fixation, each one focusing on different aspects. Therefore, the entropy method was used to calculate the weighting factors to determine the stability of different rib fracture fixation structures [28].

### 2.4. Statistical Analysis

GraphPad Prism 9 and SPSS 22 (IBM, Armonk, NY, USA) were used for statistical analysis. The results of the FEA are presented as mean ± standard deviation (mean ± SD) for normally distributed data or median (25%, 75%) for non-normally distributed data. The normality of all variables was first assessed using the Shapiro–Wilk test. For data satisfying normality, Levene’s test was further performed to evaluate the homogeneity of variances. An analysis of variance (ANOVA) was used to assess the significant differences between the fixation strategies when the data met both normality and homogeneity of variances assumptions. Conversely, the Kruskal–Wallis test was applied for between-group comparisons when the normality or homogeneity of variances was violated. The entropy method was used to score and rank the results of the biomechanical parameters for different fixation methods. A *p*-value of less than 0.05 was considered to be statistically significant.

## 3. Results

### 3.1. Entropy Score

**Figure 4:**
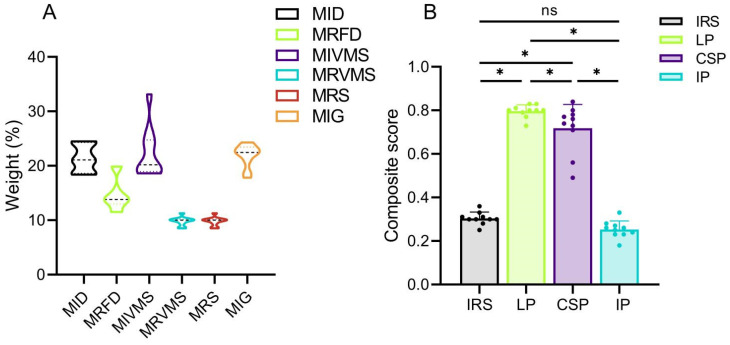
Entropy scoring method results. (**A**) Weighting coefficients for fracture stability assessment parameters; (**B**) Composite scores for different fixations. * *p* < 0.05. ns: not significant.

Biomechanical parameters were evaluated using the entropy method, and composite scores were calculated for the four fixation methods. Among the biomechanical parameters evaluated, MIVMS, MIG, and MID exhibited lower entropy values and higher information utility values and weight coefficients (Figure 4A and Table 2). In contrast, MRFD, MRVMS, and MRS exhibited higher entropy values, lower information utility values, and lower weight coefficients (*p* < 0.05). The highest and lowest composite scores were LP (0.8 ± 0.03) and IP (0.25 ± 0.04), respectively (Figure 4B and Table A3).

### 3.2. MID and MRFD

**Figure 5:**
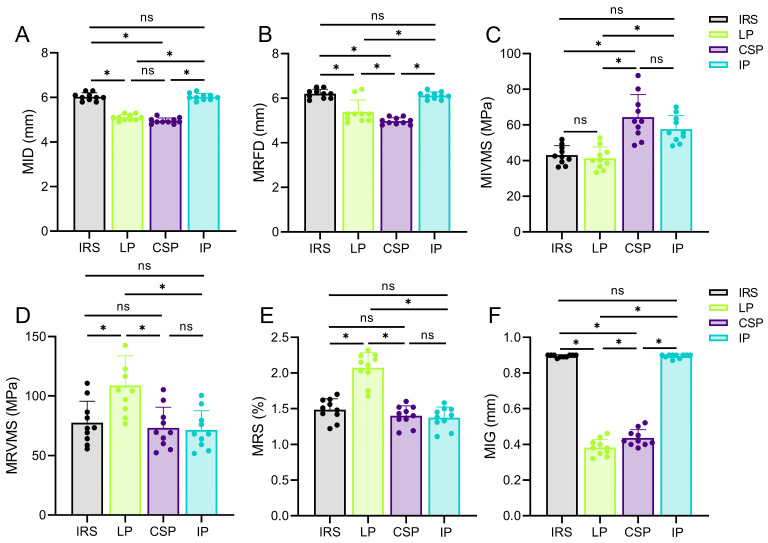
The maximal implant displacement (**A**) (MID, mm), the maximal rib displacement (**B**) (MRFD, mm), the maximal implant von Mises stress (**C**) (MIVMS, MPa), the maximal rib von Mises stress (**D**) (MRVMS, MPa), the maximal rib strain (**E**) (MRS, %), and the maximal interfragmentary gap (**F**) (MIG, mm) of four fixation structures under anterior and posterior loading. * *p* < 0.05. ns: not significant.

**Figure 6:**
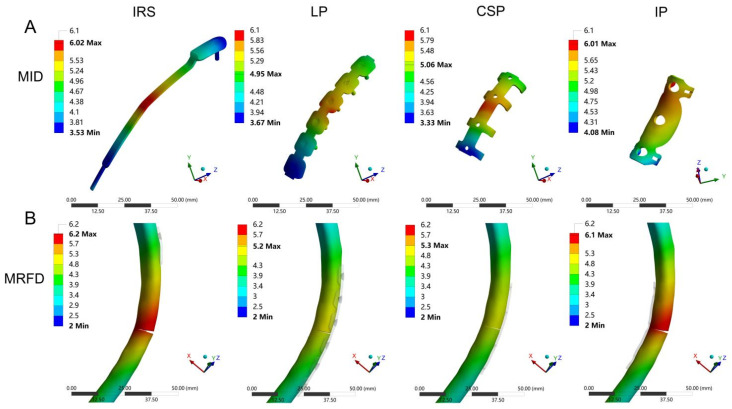
The cloud diagram of the maximal implant displacement (MID, mm) (**A**) and the maximal rib displacement (MRFD, mm) (**B**) for four fixation methods.

The MID and MRFD results of all patient-specific rib fracture fixation models in case of frontal impact are shown in Figure 5A,B and Figure 6, and Table A4. The MID and MRFD of each group were mainly concentrated in the implant–fracture line interface area. The highest and lowest MID values were observed in the IRS (6.03 mm ± 0.18 mm) and LP (4.95 ± 0.13) groups, respectively, reflecting a 17.9% reduction in MID for the LP compared with the IRS (*p* < 0.05; Figure 6A). Similar trends were observed for the MRFD. Specifically, the highest and lowest MRFD values were observed in the IRS (6.20 mm ± 0.21 mm) and LP (4.98 mm ± 0.15 mm) groups, respectively, with a 19.7% reduction in MRFD for the LP compared with the IRS (*p* < 0.05; Figure 6B).

### 3.3. MIVMS and MRVMS

**Figure 7:**
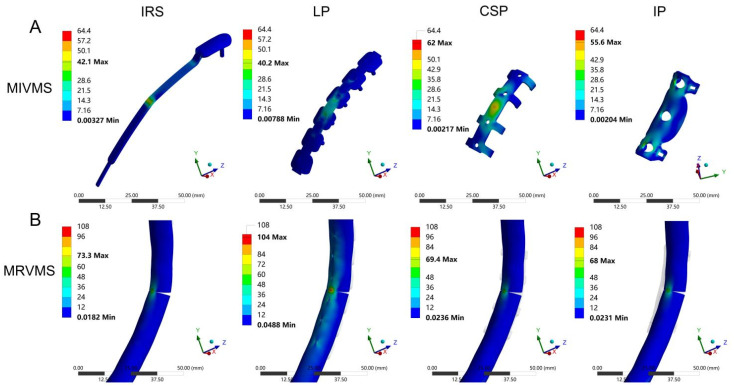
The cloud diagram of the maximal implant von Mises stress (MIVMS, MPa) (**A**) and the maximal rib von Mises stress (MRVMS, MPa) (**B**) for four fixation methods.

The VMS distributions and values of the four implants and fracture ends are shown in Figure 5C,D and Figure 7A,B, and Table A4. The MIVMS was mainly concentrated in the implant–fracture line interface area. The highest and lowest MIVMS values were observed in the CSP (64.4 MPa ± 12.6 MPa) and LP (41.3 MPa ± 6.3 MPa) groups, respectively, reflecting a 35.9% reduction in the MRVMS for the LP compared with the CSP (*p* < 0.05; Figure 5C). The MRVMS in each group was mainly concentrated in the fracture line area of the medial cortex of the rib. The highest and lowest MRVMS values were observed in the LP (108.8 MPa ± 25.1 MPa) and the IP (71.5 MPa ± 16.1 MPa) groups, respectively, with a 34.3% reduction in the MRVMS for the IP compared with the LP (*p* < 0.05; Figure 5D).

### 3.4. MRS and MIG

The distribution and values of the MRS and MIG are shown in Figure 5E,F and Table A4. The MRS was mainly concentrated in the fracture line area of the medial cortex of the rib. The highest and lowest MRS values were observed in the LP (2.07% ± 0.21%) and IP (1.37% ± 0.15%) groups, respectively, with a 33.8% reduction in the MRS for the IP compared with the LP (*p* < 0.05; Figure 5E). The MIG in each group was concentrated in the fracture line area of the lateral cortex of the rib. The highest and lowest MIG values were observed in the IRS (0.90 ± 0.01 mm) and LP (0.38 ± 0.05 mm), respectively, with a 57.8% reduction in the MIG for the LP compared with the IRS (*p* < 0.05; Figure 5F).

## 4. Discussion

A single rib fracture may lead to chronic pain, decreased respiratory function, and reduced long-term quality of life [29]. Retrospective studies and randomized controlled trials have demonstrated that the SSRF improves patient clinical outcomes [30]. Currently, the biomechanical properties of implants for treating rib fractures and the advantages of different fixation methods remain unclear. Appropriate implants foster appropriate mechanical conditions for fracture healing and provide sufficient stability to alleviate pain and dysfunction. Therefore, studying the biomechanical properties of different fixation methods from a biomechanical perspective is crucial to provide a reference for selecting appropriate implants for rib fractures.

To the best of our knowledge, this is the first comprehensive biomechanical study of the SSRF to compare six biomechanical parameters of 10 patient-specific models stabilized using four different implants. Previous studies have assessed the biomechanical stability of rib fracture fixation using parameters such as implant and bone displacements and stresses [31]. Notably, the variability of these outcome measures must be recognized, particularly for parameters such as the MIVMS and MRVMS, which exhibit significant standard errors [32]. Therefore, biomechanical outcomes may vary among patients, even with the identical fixation structures. These variations stem from individual differences in bone mineral density and anatomical morphology. Consequently, constructing patient-specific FE models with a substantial number of samples is crucial to significantly eliminate the impact of individual differences on experimental outcomes. A desirable performance of a model is characterized by low values for each of the six biomechanical parameters (MID, MRFD, MIVMS, MRVMS, MRS, and MIG). The entropy method enables the synthesis of all the biomechanical parameter results and facilitates the comparison of the stability among various fixation modalities, while identifying the significant biomechanical parameters [32]. Our comprehensive scoring results, based on independent biomechanical parameters, demonstrated better biomechanical stability with the LP compared with the other fixation methods for treating rib fractures.

**Figure A1:**
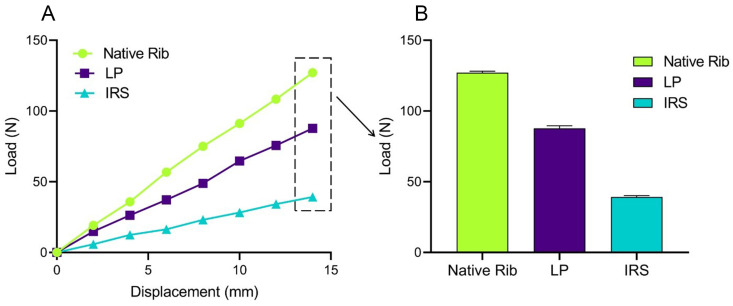
The stiffness results of the native ribs, locking plates (LP), and intramedullary rib splints (IRS) used for validation of model validity: (**A**) the displacement-load curves, with the slopes of the curves representing the stiffnesses of the groups; (**B**) the load values at displacements of up to 14 mm for each group.

We also verified the effectiveness of the simulation model by comparing the FE simulation results with those of the biomechanical studies reported in the literature [33,34]. Thus, under identical experimental conditions, the patient-specific rib stiffness values obtained from the FEA (Figure A1)—native (9.08 ± 0.08 N/mm), LP fixation (6.27 ± 0.12 N/mm), and IRS fixation (2.81 ± 0.06 N/mm)—were consistent with the biomechanical data reported by Bottlang et al. (native 10.0 ± 6.0 N/mm, LP fixation 7.0 ± 4.0 N/mm, and IRS fixation 2.0 ± 1.0 N/mm), with a standard deviation of ±1, indicating the suitability of our model construction method for further analysis.

Implant and bone displacements are typical outcome parameters for the FEA and have been widely evaluated in in-silico studies [31,35]. Our results revealed higher fixation stabilities with the CSP and LP, owing to their lower MID and MRFD values. In contrast, the IRS and IP fixation exhibited a lower stability compared with previous findings [20,36]. This may be related to the anatomical characteristics of the ribs, as the maximum bending moment occurs in the lateral region of the rib under an anteroposterior load. The lateral cortical fixation techniques of the CSP and LP enhanced the lateral cortical stability of the ribs, resulting in smaller bone and implant displacements during rib collisions. Therefore, the CSP and LP were more reliable in terms of fixation strength. Furthermore, in addition to reduction and firm fixation, the blood supply to the fracture end is critical for fracture healing [37]. Compared with the CSP, smaller bone and implant displacements were observed with the LP. The CSP may compress the intercostal nerves and blood vessels during the fracture end fixation, causing chest pain and affecting the blood supply. Typically, the LP does not require freeing the vascular and nerve bundles at the lower edge of the ribs during implantation, offering certain advantages in preserving the blood supply [38]. Therefore, a LP is an appropriate implant for the treatment of rib fractures, whether for fixation stability or protection of blood supply.

Peak stress is associated with static yield or cyclic fatigue failure, and fixation failure often begins at the stress concentration site [39]. In the present study, the MIVMS of the LP was lower than that of the other groups, indicating more uniform stress distribution in the LP with a lower risk of fixation failure. This may be associated with the stress-transfer modes of the different fixation methods. The LP acts as a stress-transfer bridge between the fractured ends of the anterior and posterior ribs. The stress is evenly shared by the LP and the screws, thereby reducing bone stress and displacement [34]. The CSP stabilizes the fracture ends via the clasp mechanism of the circlip, counteracting the bending moment generated by the anteroposterior load of the ribs and reducing fixation stress [38]. The IRS restores the continuity of rib fractures via an intramedullary implant for elastic fixation [33]. The IP restores the load transfer by enhancing the medial cortical stability, effectively reducing the MRVMS value in the medial cortex [40]. Existing implants are sufficient for maintaining the stability of the chest wall under normal physiological breathing conditions. Meanwhile, accident-induced fractures around implants during postoperative rehabilitation are a significant concern. Therefore, suitable fixation devices should prevent new fractures in vulnerable areas of the chest wall when subjected to external forces. The stress distribution results show that the LP fixation structure not only firmly stabilizes the fracture ends but also effectively reduces the stress on the fixation structure and provides better stability and safety in some cases, such as minor impacts.

Bone strain is a biomechanical parameter commonly used to predict bone failure. By measuring the axial and shear components at the end of the fracture fragment, the interfragmentary strain and stability of the fracture fixation can be evaluated in a normalized manner, which is of great predictive significance for fracture healing [41,42]. Our findings revealed that the CSP and LP exhibited lower MIG values than the IRS and IP when subjected to a frontal collision. This implies that the intramedullary and medial rib cortical fixations are less stable than lateral rib cortical fixation under extreme conditions. Elkins et al. [43], through the FEA of patient-specific fracture healing based on radiographic data, demonstrated that appropriate axial micromotion at the fracture site promotes fracture healing, whereas excessive separation motion inhibits fracture healing. Under the mechanical effect of the implant, the MIG at the fracture site exhibited a state of “relative stability”. As the fracture healing progresses, the callus gradually hardens, and the variable gap decreases, ultimately restoring the load transmission through the fracture [44]. In case of an external impact, lateral rib cortical fixation methods, such as the LP and CSP, exhibit lower MIG values, indicating a greater potential for promoting fracture healing. However, further validation of this finding is warranted using clinical studies with large sample sizes. In addition, the MIG value of the CSP was higher than that of the LP, which is consistent with the results of biomechanical experiments conducted by Huang et al. [38]. As long as the fracture fixation provides sufficient stability, more flexible structures can be beneficial for fracture healing [45]. Therefore, in addition to the LP, lateral cortical fixation strategies, such as the CSP, also appear to exhibit unique advantages in treating rib fractures.

The present study is the first to use FEA to comprehensively evaluate the biomechanical effects of four different fixation methods on the stability after rib fracture fixation. Nevertheless, this study has some limitations. First, despite our efforts to mitigate the impact of variations in rib morphology and material properties on experimental outcomes by increasing the sample size, the number of patient samples remains limited. Consequently, the trends observed in this study may not be applicable to all patients. Second, not including the cartilage, thoracic organs, and vertebrae in our FEA model poses challenges to our ability to fully evaluate the effect of fixation structures on the overall stability of the chest wall. Third, the scope of this study was confined to the assessment of a single loading condition and, as such, neglects other clinically relevant conditions, such as thoracic forces on the ribs, sternum, and costal cartilage during respiration.

## 5. Conclusions

Our results indicate that the MIVMS, MIG, and MID are critical biomechanical parameters for evaluating rib fracture fixation stability. The patient-specific finite element method entropy score revealed the mechanical stability of the different rib fracture fixation methods. Notably, in extreme cases, lateral cortical fixation of a fractured rib may substantially contribute to its structural stability.

## Figures and Tables

**Table 1:** Element information consisting of finite element models.

Finite Element Models	IRS	LP	CSP	IP
Number of nodes	807,666	826,224	812,666	820,248
Number of elements	518,272	530,978	523,827	528,300
Size of element, (mm)				
Mean	0.82	0.83	0.84	0.83
Maximum	1.00	1.00	1.00	1.00
Minimum	3.47 × 10^−2^	3.70 × 10^−2^	3.72 × 10^−2^	2.96 × 10^−2^

**Table 2:** Entropy value, utility value, and weight of biomechanical parameters.

Parameters	Entropy Value (e)	Utility Value (d)	Weighting Coefficient (%)
MID	0.54 ± 0.06	0.46 ± 0.06	21.4 ± 2.4
MRFD	0.68 ± 0.07	0.32 ± 0.07	14.7 ± 2.9
MIVMS	0.52 ± 0.14	0.48 ± 0.14	22.4 ± 4.6
MRVMS	0.79 ± 0.01	0.21 ± 0.01	9.8 ± 0.8
MRS	0.79 ± 0.01	0.21 ± 0.01	9.8 ± 0.8
MIG	0.53 ± 0.03	0.47 ± 0.03	21.9 ± 2.1

## Data Availability

The raw data supporting the conclusions of this article will be made available by the authors, without undue reservation.

## Abbreviations

CSP	Claw-shape plate
CT	Computed tomography
FEA	Finite element analysis
IRS	Intramedullary rib splint
LP	Locking plate
MID	Maximum implant displacement
MIG	Maximum interfragmentary gap
MIVMS	Maximum implant von Mises stress
MRFD	Maximum rib fracture displacement
MRS	Maximum rib strain
MRVMS	Maximum rib von Mises stress
SSRF	Surgical stabilization of rib fractures

## Appendix A

**Table A1:** Baseline information for patients.

Patient	Age	Gender	BMI (kg/cm^2^)	Smoking History	Rib Neck Length ^1^ (mm)	Rib Chord Length ^2^ (mm)	Rib Curve Length ^3^ (mm)
1	27	F	33.4	Yes	35.2	186.1	318.3
2	38	F	32.1	No	37.4	188.2	320.5
3	50	F	26.8	Yes	34.4	178.8	300.1
4	47	F	29.1	Yes	32.1	184.3	313.6
5	35	F	24.5	Yes	34.8	190.2	330.4
6	68	M	19.8	No	34.1	177.6	299.8
7	72	M	21.2	No	32.7	175.4	298.7
8	49	M	26.1	No	33.6	182.6	308.4
9	39	M	21.2	No	31.1	183.2	311.9
10	66	M	22.6	No	30.7	179.8	300.8

^1^ Rib neck length: Distance from the medial edge of the rib head to the most lateral border of the articular facet of the rib tubercle. ^2^ Rib chord length: Linear distance from the most lateral aspect of the rib head to the costo-chondral junction. ^3^ Rib Curve Length: Curved distance from the most lateral aspect of the rib head to the costo-chondral junction.

**Table A2:** Mesh convergence analysis of maximum von Mises stress in relation to mesh size for implant and bone models in locking plate fixation structures.

Mesh Size (mm)	0.4	0.6	0.8	1
MIVMS (MPa)	47.9 ± 7.1	43.2 ± 6.8	41.3 ± 6.3	40.8 ± 5.4
MRVMS (MPa)	120.8 ± 27.4	111.2 ± 25.3	108.8 ± 25.1	106.2 ± 22.4
Computing time (s)	7215.8 ± 124.3	1355.7 ± 64.2	408.1 ± 44.5	390.5 ± 40.2

**Table A3:** Composite scores for four fixation methods.

	Patient 1	Patient 2	Patient 3	Patient 4	Patient 5	Patient 6	Patient 7	Patient 8	Patient 9	Patient 10	Composite Score
IRS	0.36	0.30	0.31	0.30	0.25	0.28	0.31	0.30	0.30	0.33	0.30 ± 0.03
LP	0.80	0.73	0.80	0.80	0.83	0.79	0.83	0.80	0.81	0.77	0.80 ± 0.03
CSP	0.71	0.84	0.74	0.78	0.49	0.80	0.73	0.77	0.56	0.76	0.72 ± 0.11
IP	0.23	0.26	0.23	0.25	0.24	0.28	0.18	0.26	0.27	0.33	0.25 ± 0.04

**Table A4:** Results of different biomechanical parameters in FEA.

	IRS	LP	CSP	IP
MID (mm)	6.03 ± 0.18	4.95 ± 0.13	5.10 ± 0.13	6.03 ± 0.15
MRFD (mm)	6.20 ± 0.21	4.98 ± 0.15	5.39 ± 0.53	6.12 ± 0.16
MIVMS (MPa)	43.1 ± 5.2	41.3 ± 6.3	64.4 ± 12.6	57.7 ± 7.5
MRVMS (MPa)	77.5 ± 18.1	108.8 ± 25.1	73.2 ± 17.3	71.5 ± 16.1
MRS (%)	1.48 ± 0.15	2.07 ± 0.21	1.40 ± 0.14	1.37 ± 0.15
MIG (mm)	0.90 ± 0.01	0.38 ± 0.05	0.44 ± 0.05	0.89 ± 0.01
